# Supplementary material for: Properly Substituted Cyclic Bis-(2-bromobenzylidene) Compounds Behaved as Dual p300/CARM1 Inhibitors and Induced Apoptosis in Cancer Cells
Source: Molecules. 2020 Jul 8;25(14):3122. doi: 10.3390/molecules25143122 (PMC7397249; doi:10.3390/molecules25143122)

Rossella Fioravanti, Stefano Tomassi, Elisabetta Di Bello, Annalisa Romanelli, Andrea Maria Plateroti, Rosaria Benedetti, Mariarosaria Conte, Ettore Novellino, Lucia Altucci, Sergio Valente*, Antonello Mai*

Properly Substituted Cyclic Bis-(2-bromobenzylidene) Compounds Behaved as Dual p300/CARM1 Inhibitors and Induced Apoptosis in Cancer Cells

Supplementary material

Figure S1. Western blot analyses of the levels of acetyl-H3K9/14 and EZH2 in NB4 cells treated with **4d-n** at 5 μM for 30 h. H4 and ERK were used for equal loading.

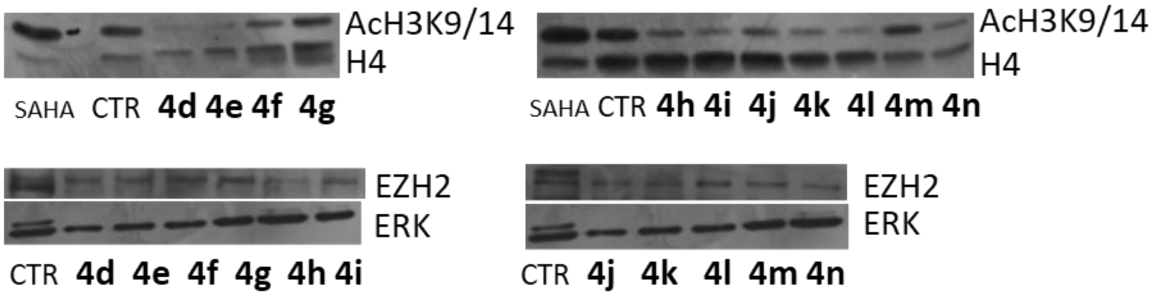

1-benzyl-3,5-bis((E)-2-bromobenzylidene)piperidin-4-one, ^{1}H, DMSO

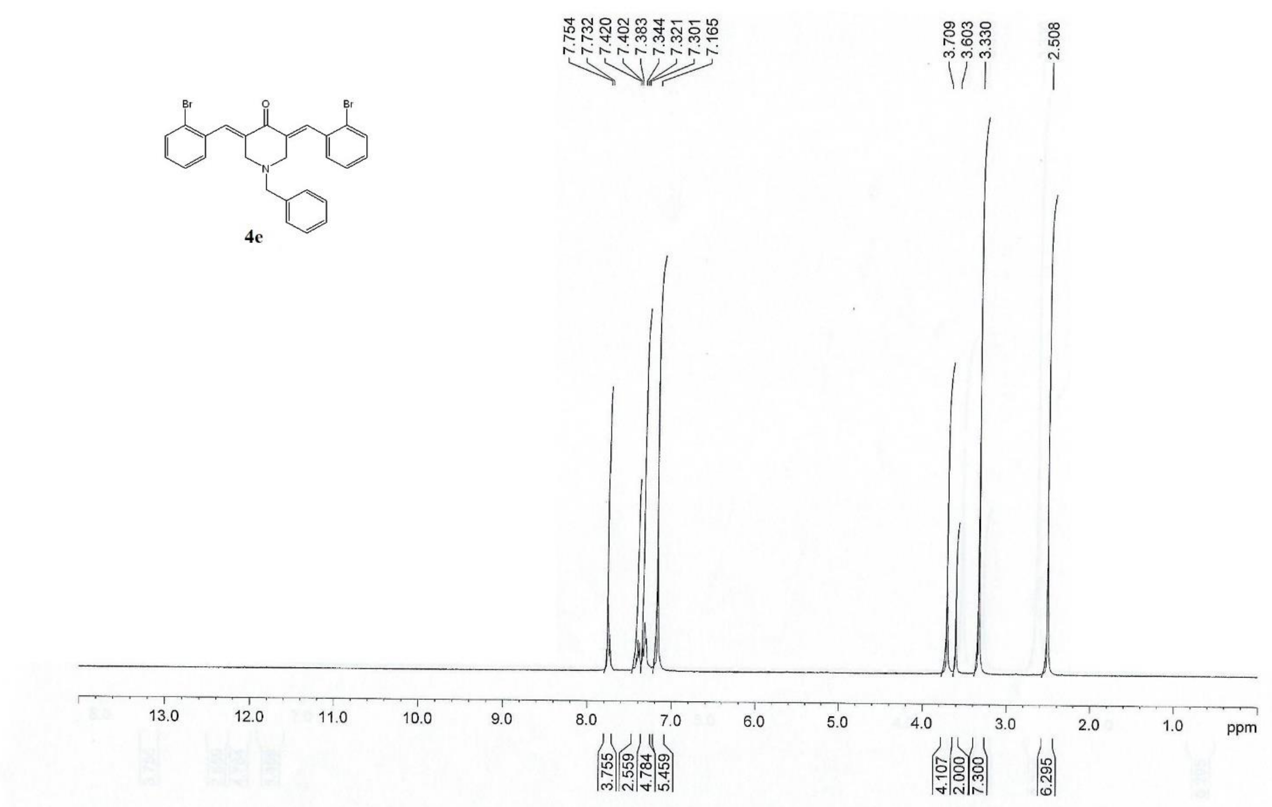

1-benzyl-3,5-bis((E)-2-bromobenzylidene)piperidin-4-one, ^{13}C, DMSO

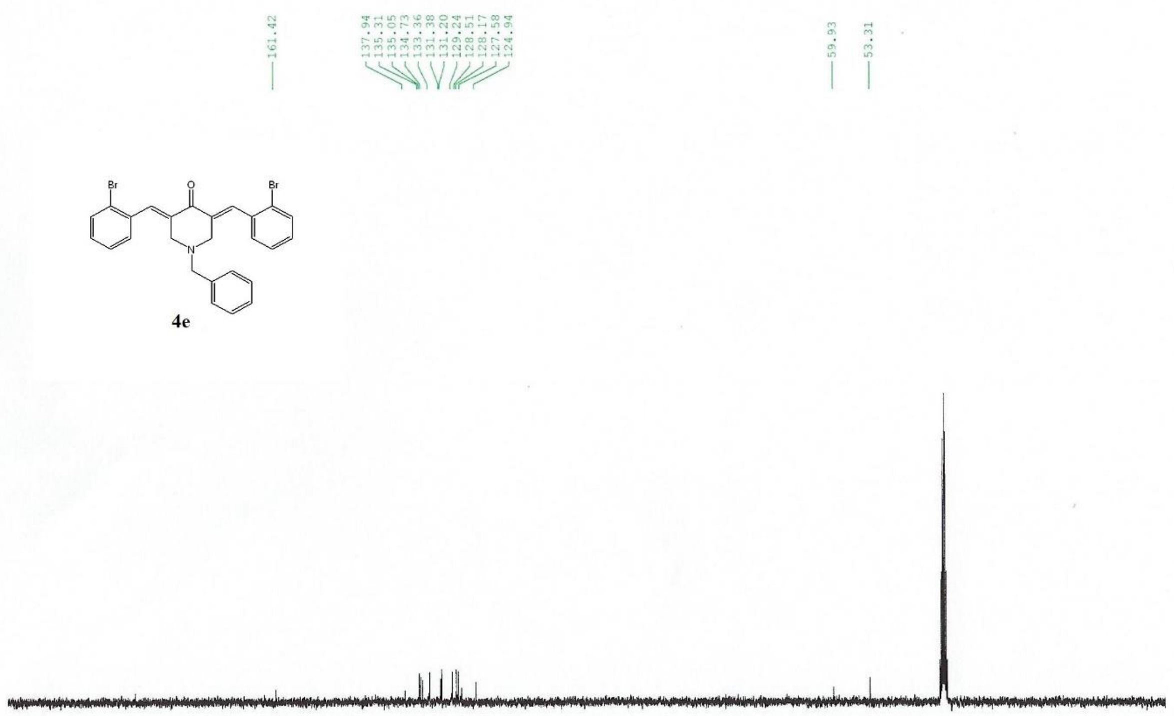

3,5-bis((E)-2-bromobenzylidene)-1-phenethylpiperidin-4-one, ^1H, $CDCl_3$

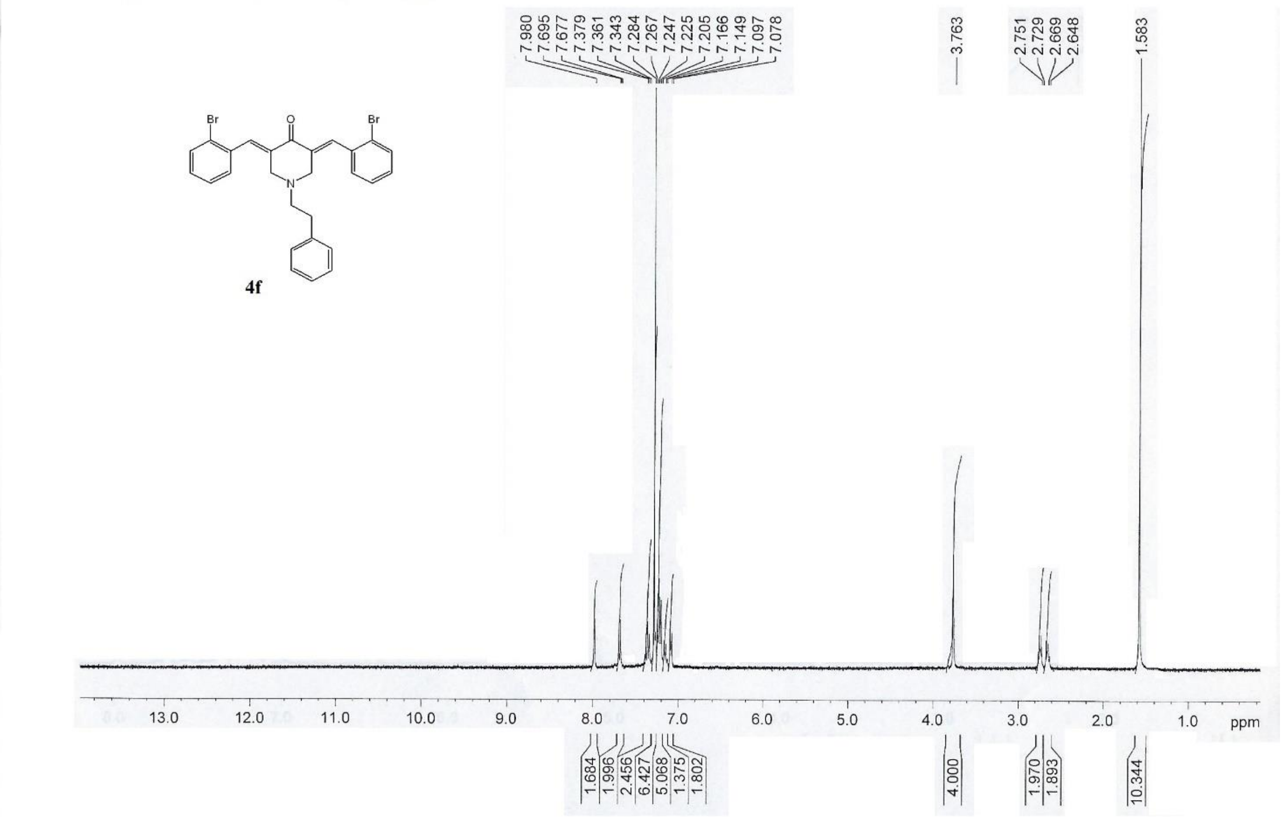

3,5-bis((E)-2-bromobenzylidene)-1-(3-phenylpropyl)piperidin-4-one, ^1H, $CDCl_3$

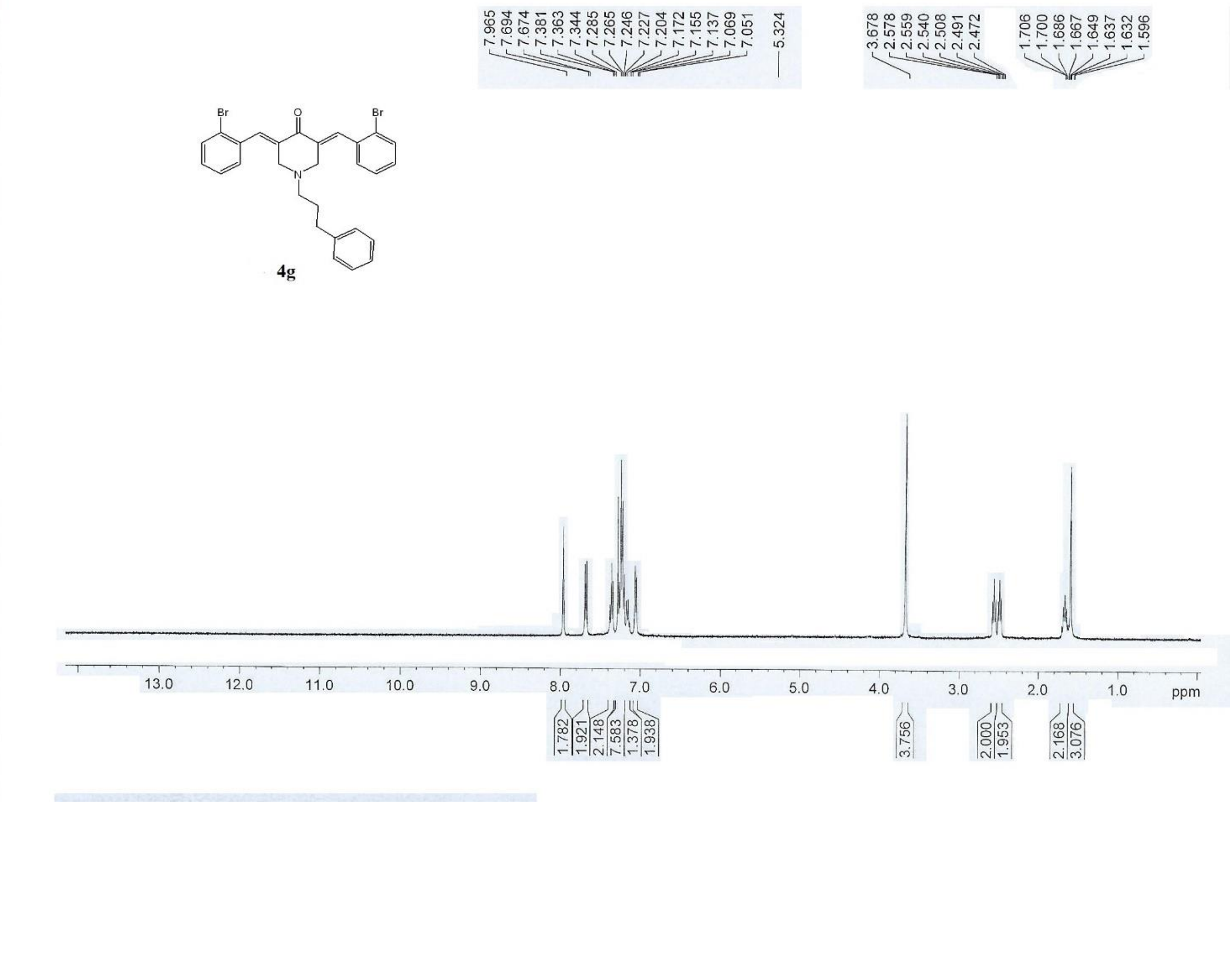

3,5-bis((E)-2-bromobenzylidene)-1-(2-oxo-2-phenylethyl)piperidin-4-one, ^{1}H, $CDCl_3$

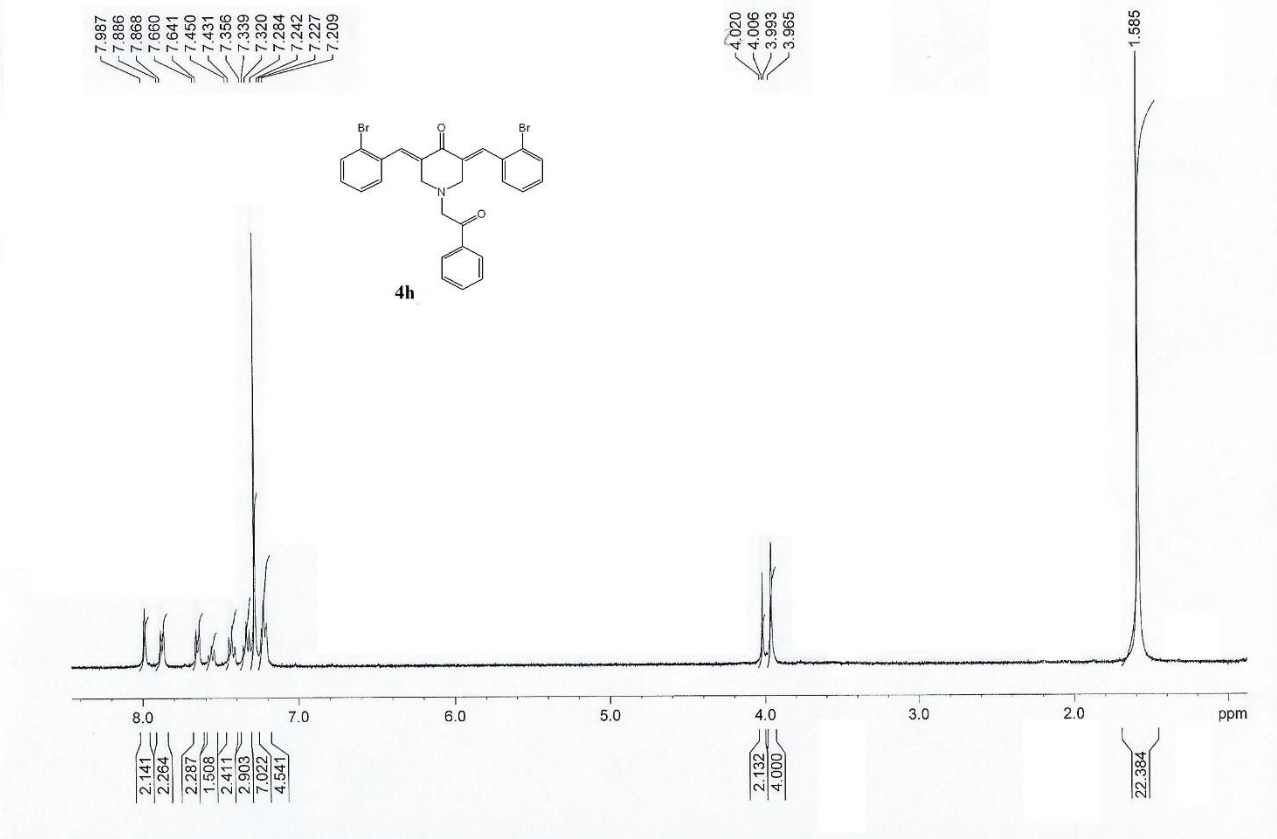

3,5-bis((E)-2-bromobenzylidene)-1-(2-oxo-2-phenylethyl)piperidin-4-one, ^{13}C, DMSO

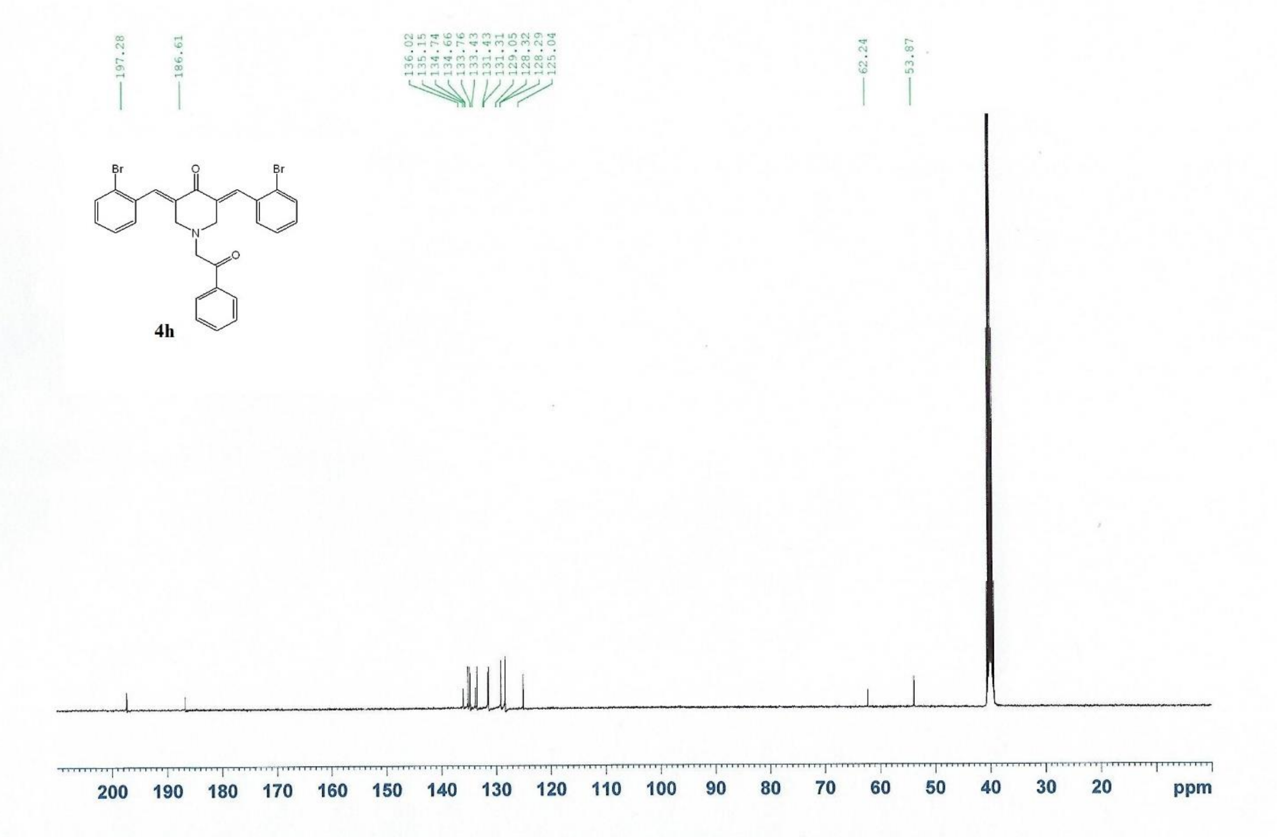

3,5-bis((E)-2-bromobenzylidene)-1-(3-oxo-3-phenylpropyl)piperidin-4-one, ^{1}H, CDCl$_3$

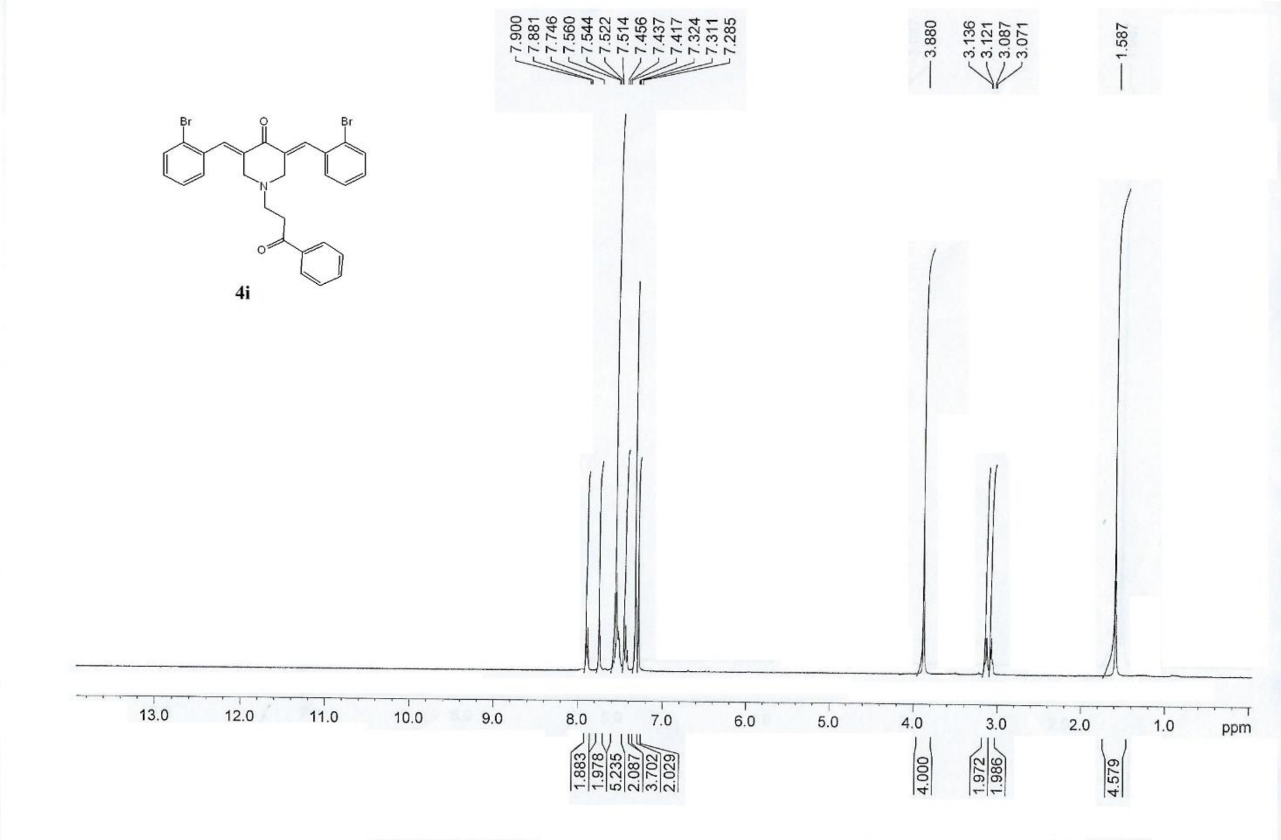

3,5-bis((E)-2-bromobenzylidene)-1-(4-oxo-4-phenylbutyl)piperidin-4-one, ^{1}H, CDCl$_3$

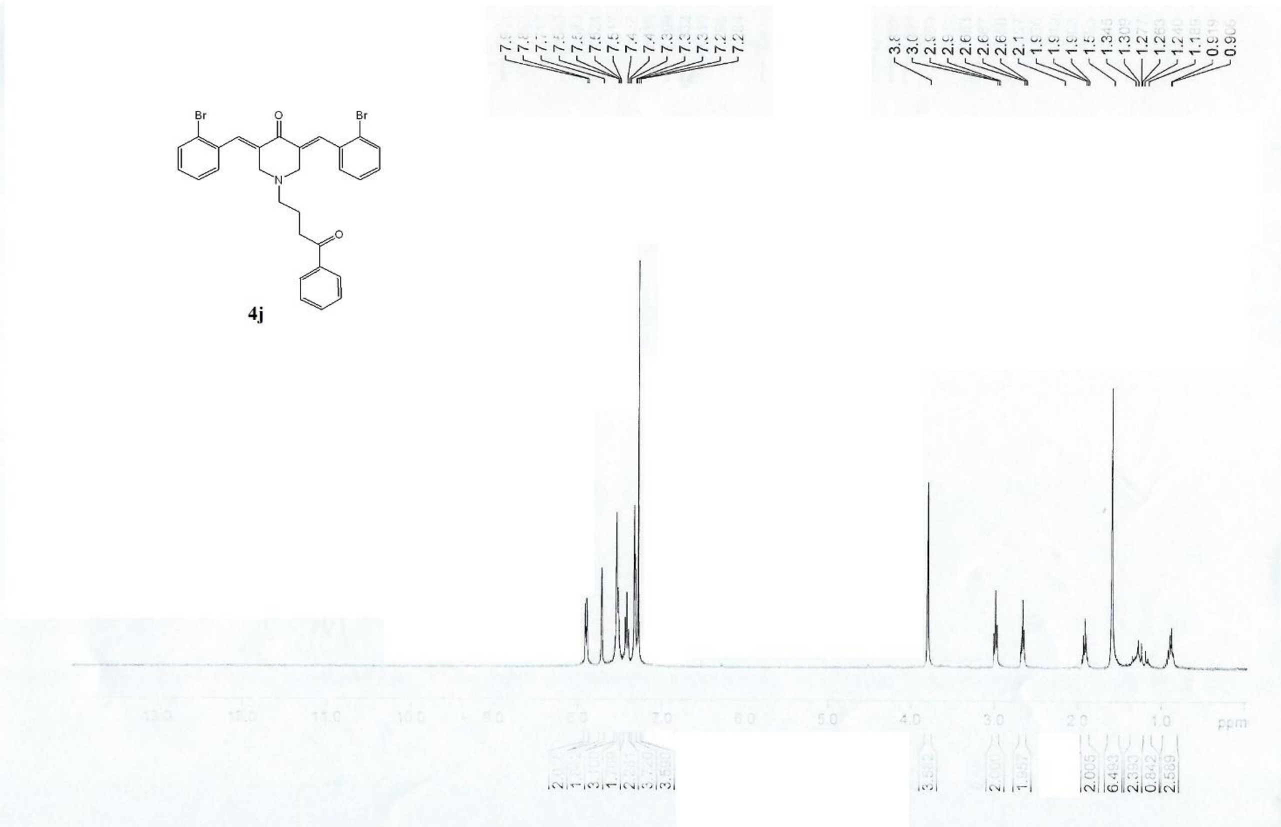

1-benzoyl-3,5-bis((E)-2-bromobenzylidene)piperidin-4-one, ^1H, $CDCl_3$

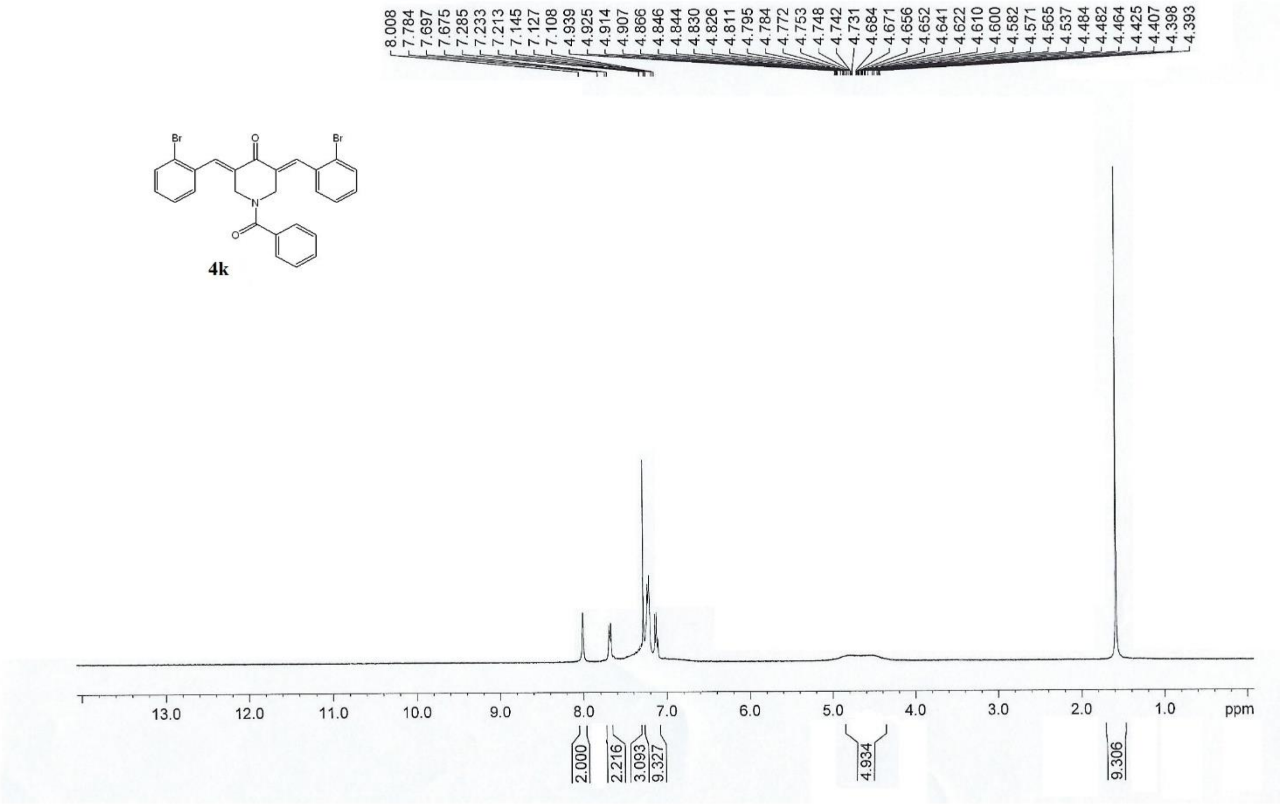

3,5-bis((E)-2-bromobenzylidene)-1-(2-phenylacetyl)piperidin-4-one, ^1H, $CDCl_3$

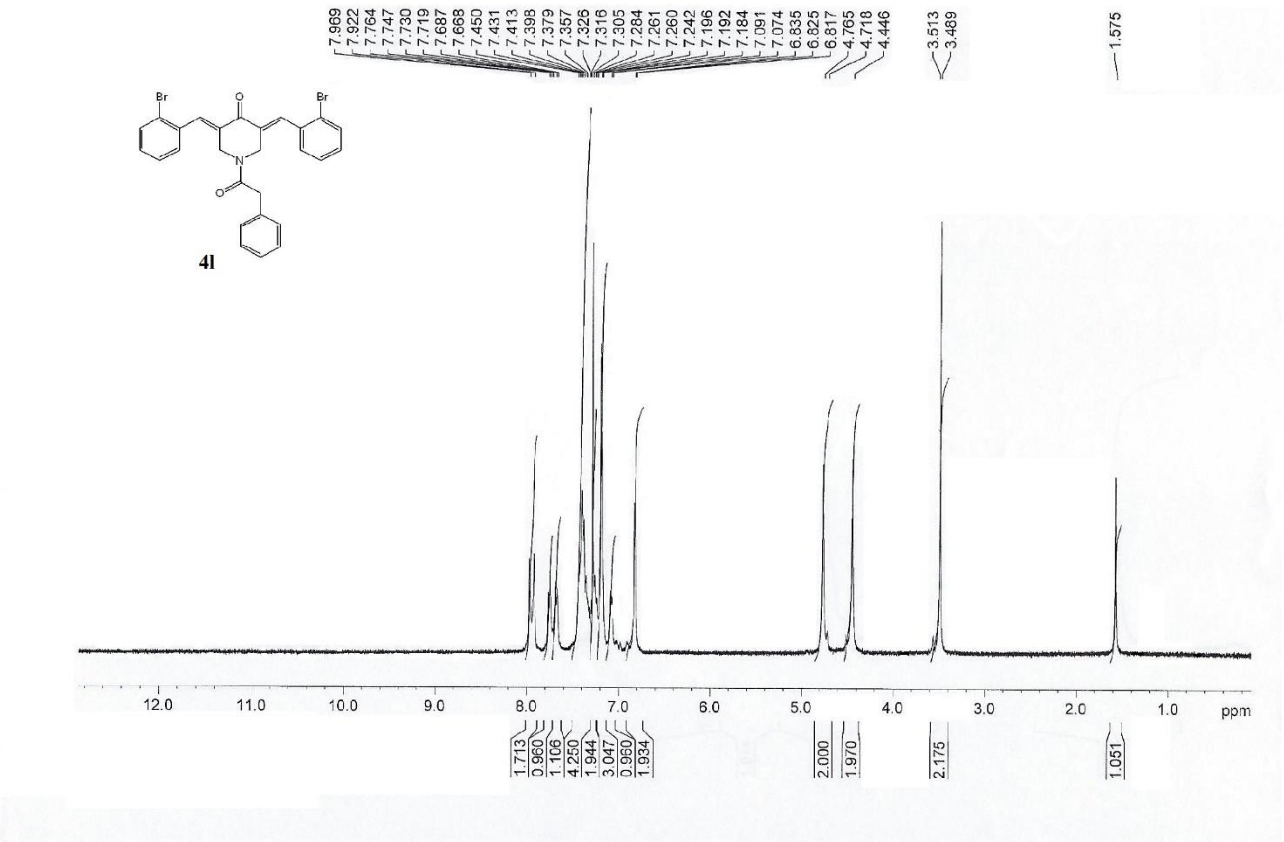

3,5-bis((E)-2-bromobenzylidene)-1-(2-phenylacetyl)piperidin-4-one, ^{13}C, DMSO

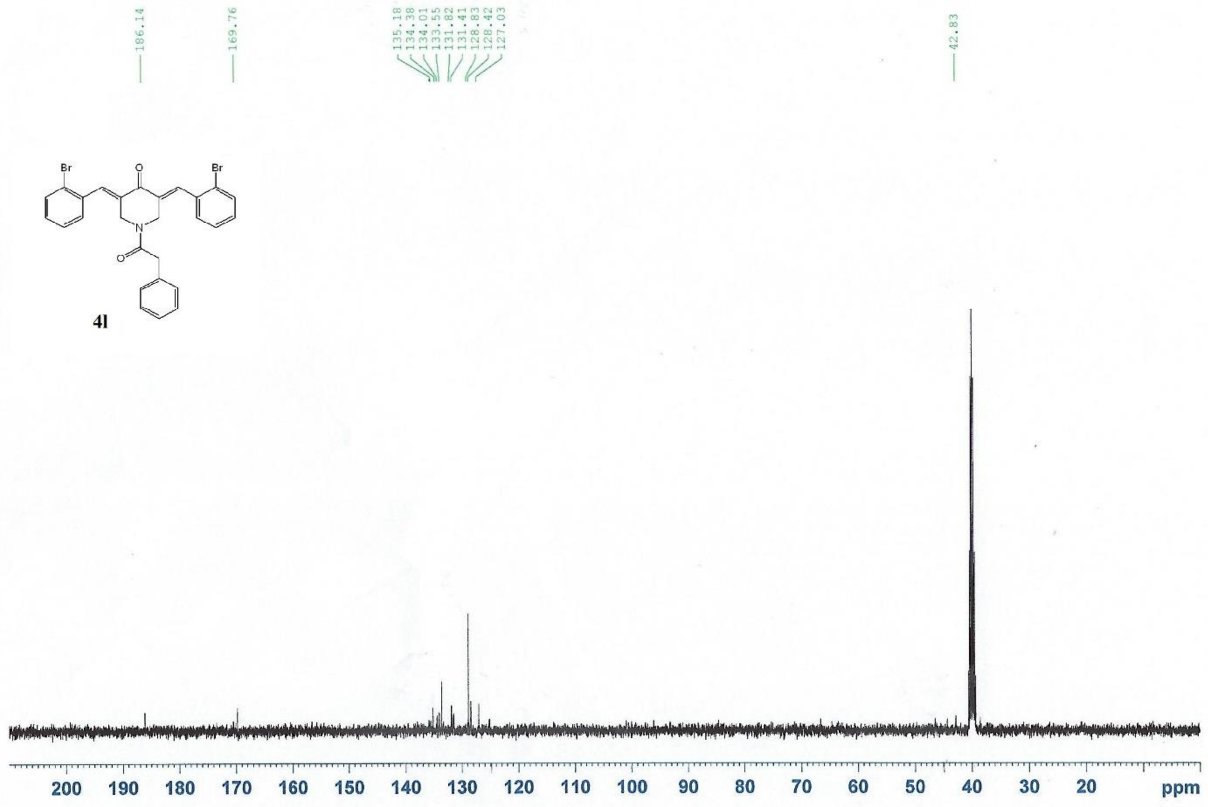

3,5-bis((E)-2-bromobenzylidene)-1-(3-phenylpropanoyl)piperidin-4-one, ^{1}H, $CDCl_3$

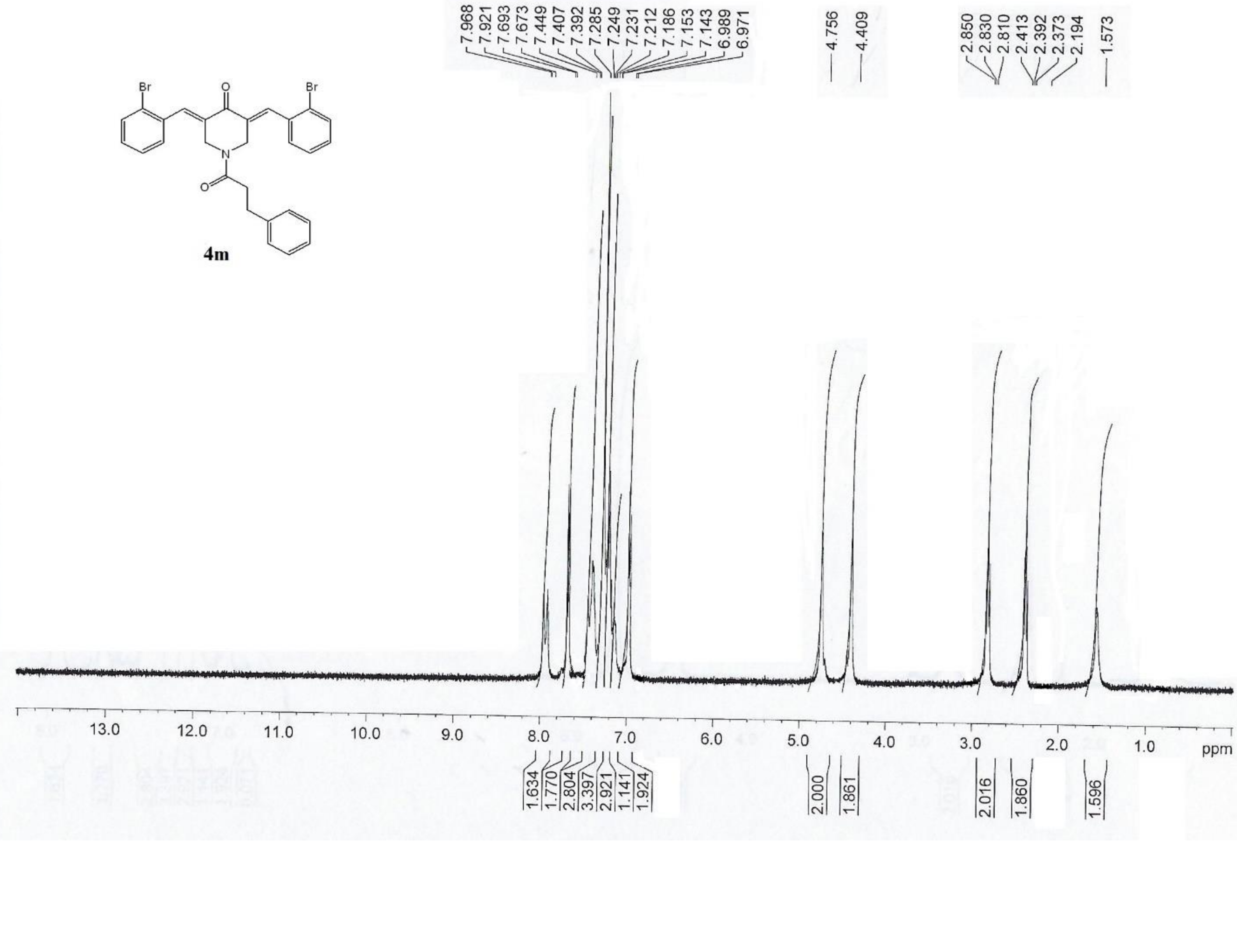

3,5-bis((E)-2-bromobenzylidene)-1-cinnamoylpiperidin-4-one, ^{1}H, $CDCl_3$

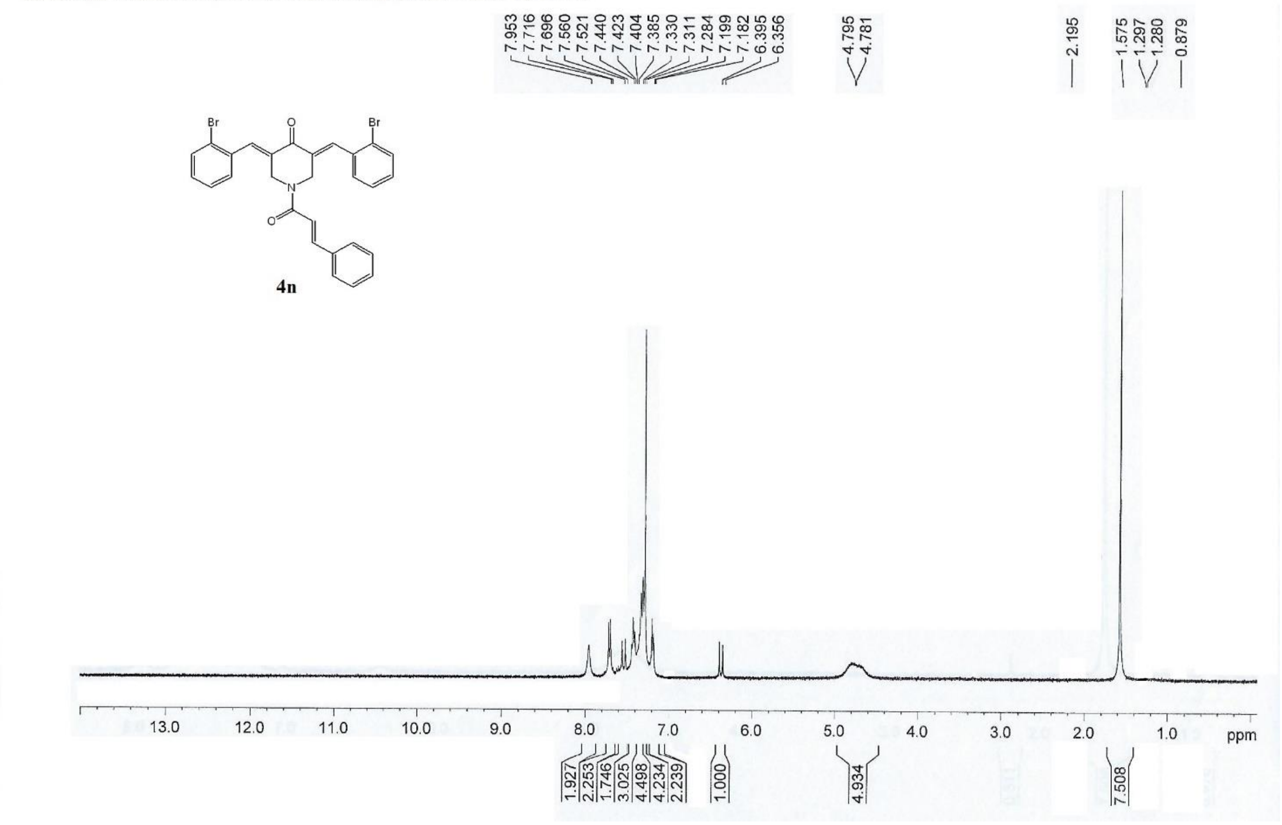

Supplement: Supplementary file 1 [file molecules-25-03122-s001.pdf]
